# Iron-regulated assembly of the cytosolic iron–sulfur cluster biogenesis machinery

**DOI:** 10.1016/j.jbc.2022.102094

**Published:** 2022-05-30

**Authors:** Xiaorui Fan, William D. Barshop, Ajay A. Vashisht, Vijaya Pandey, Stephanie Leal, Shima Rayatpisheh, Yasaman Jami-Alahmadi, Jihui Sha, James A. Wohlschlegel

**Affiliations:** 1 Department of Biological Chemistry, David Geffen School of Medicine, University of California, Los Angeles, California, USA; 2 Molecular Biology Institute, University of California, Los Angeles, California, USA

**Keywords:** iron–sulfur protein, metalloprotein, protein–protein interaction, proteomics, redox regulation, cytosolic iron–sulfur cluster assembly (CIA), CIA, cytosolic iron–sulfur cluster assembly, CIAO1, probable cytosolic iron-sulfur protein assembly protein CIAO1, CIAO2B, cytosolic iron-sulfur assembly component 2B, CIAO3, cytosolic iron-sulfur assembly component 3, DFO, deferoxamine, FAC, ferric ammonium citrate, ISC, mitochondrial iron–sulfur cluster assembly, MMS19, MMS19 nucleotide excision repair protein homolog, NUBP1, nucleotide-binding protein 1, NUBP2, nucleotide-binding protein 2, PRM, parallel reaction monitoring, ROS, reactive oxygen species

## Abstract

The cytosolic iron–sulfur (Fe-S) cluster assembly (CIA) pathway delivers Fe-S clusters to nuclear and cytosolic Fe-S proteins involved in essential cellular functions. Although the delivery process is regulated by the availability of iron and oxygen, it remains unclear how CIA components orchestrate the cluster transfer under varying cellular environments. Here, we utilized a targeted proteomics assay for monitoring CIA factors and substrates to characterize the CIA machinery. We find that nucleotide-binding protein 1 (NUBP1/NBP35), cytosolic iron–sulfur assembly component 3 (CIAO3/NARFL), and CIA substrates associate with nucleotide-binding protein 2 (NUBP2/CFD1), a component of the CIA scaffold complex. NUBP2 also weakly associates with the CIA targeting complex (MMS19, CIAO1, and CIAO2B) indicating the possible existence of a higher order complex. Interactions between CIAO3 and the CIA scaffold complex are strengthened upon iron supplementation or low oxygen tension, while iron chelation and reactive oxygen species weaken CIAO3 interactions with CIA components. We further demonstrate that CIAO3 mutants defective in Fe-S cluster binding fail to integrate into the higher order complexes. However, these mutants exhibit stronger associations with CIA substrates under conditions in which the association with the CIA targeting complex is reduced suggesting that CIAO3 and CIA substrates may associate in complexes independently of the CIA targeting complex. Together, our data suggest that CIA components potentially form a metabolon whose assembly is regulated by environmental cues and requires Fe-S cluster incorporation in CIAO3. These findings provide additional evidence that the CIA pathway adapts to changes in cellular environment through complex reorganization.

Iron–sulfur (Fe-S) clusters are ubiquitous cofactors utilized by all realms of life, among which [2Fe-2S] and [4Fe-4S] clusters are the most commonly found in biological systems (1). These cofactors play a role in maintaining protein stability, as well as regulating subcellular localization and enzymatic activity (2, 3, 4). Being redox sensitive, these clusters also serve as redox centers to facilitate electron transfer. The redox states of Fe-S clusters change in response to environmental stimuli, which provides an additional layer of regulation of protein function (1, 4). In eukaryotic organisms, the biogenesis of Fe-S clusters is highly compartmentalized with distinct branches of the biogenesis pathway responsible for the maturation of mitochondrial and extramitochondrial Fe-S proteins (5, 6). The maturation of extramitochondrial Fe-S proteins is facilitated specifically by the cytosolic Fe-S cluster biogenesis pathway (CIA). The CIA pathway is associated with a plethora of cellular processes, including cell proliferation, DNA damage repair, nonsense-mediated decay, apoptosis, and microtubule-based processes such as ciliogenesis (2, 7, 8, 9, 10, 11). Deregulation of CIA components and substrates has also been linked to numerous human diseases (5, 12, 13).

The maturation of cytosolic Fe-S proteins is a multistep process that is tightly regulated. In human cells, bioavailable iron is delivered for [2Fe-2S] cluster biogenesis by poly(rC)-binding protein 1 (PCBP1) to the chaperone consisting of the BolA-like protein 2 (BOLA2) and glutaredoxin-3 (GLRX3) (14). [4Fe-4S] clusters are first assembled on the CIA scaffold complex composed of nucleotide-binding protein 1 (NUBP1) and nucleotide-binding protein 2 (NUBP2) (11, 15). This step requires an unknown sulfur containing compound that is produced by the mitochondrial Fe-S cluster biogenesis (ISC) machinery and transported to the cytosol through the mitochondrial inner membrane protein ABCB7. This transiently bound [4Fe-4S] cluster is then transferred to the cluster carrier protein cytosolic iron–sulfur assembly component 3 (CIAO3) and eventually incorporated into apoprotein substrates through the activity of the CIA targeting complex composed of MMS19 nucleotide excision repair protein homolog (MMS19); probable cytosolic iron-sulfur protein assembly protein CIAO1 (CIAO1); cytosolic iron-sulfur assembly component 2B (CIAO2B) (2, 16).

Although the crosstalk has been extensively documented in numerous organisms between Fe-S cluster biogenesis and the cellular environment such as intracellular iron and oxygen levels, evidence supporting this idea is only just beginning to emerge in humans (1, 4). The availability of bioavailable iron was recently shown to regulate cytosolic [2Fe-2S] cluster biogenesis by controlling the association of BOLA2 and GLRX3 (17). Additionally, the maturation of specific extramitochondrial Fe-S proteins involved in DNA repair and iron homeostasis are regulated by iron and oxygen availability (18, 19). Despite these advances, however, the mechanisms underlying much of this regulation are still unknown.

In this work, we developed a targeted proteomics assay that monitor proteins in the CIA pathway. Using this assay, we were able to detect the association of the CIA targeting complex (MMS19, CIAO1, and CIAO2B) and CIA substrates (DNA2, POLD1, CDKAL1, and ERCC2) with the CIA scaffold complex component NUBP2. We also find that the interaction of CIAO3 with the CIA scaffold complex is regulated by acute changes in cellular environment, including changes in the labile iron pool, exposure to reactive oxygen species (ROS), and changes in oxygen tension. The interaction of CIAO3 with the CIA targeting complex, although minimally affected by these acute environmental changes, is dependent on Fe-S cluster binding by CIAO3. CIAO3 mutants that are defective in Fe-S cluster binding display impaired association with the rest of the CIA machinery. Together, these data suggest the formation of CIA metabolon composed of the CIA scaffold complex, CIAO3, the CIA targeting complex and CIA substrates. The metabolon assembly is dynamic and regulated by environmental cues, possibly through altering Fe-S clusters in CIAO3.

## Results

### Formation of the CIA metabolon

**Figure 1:**
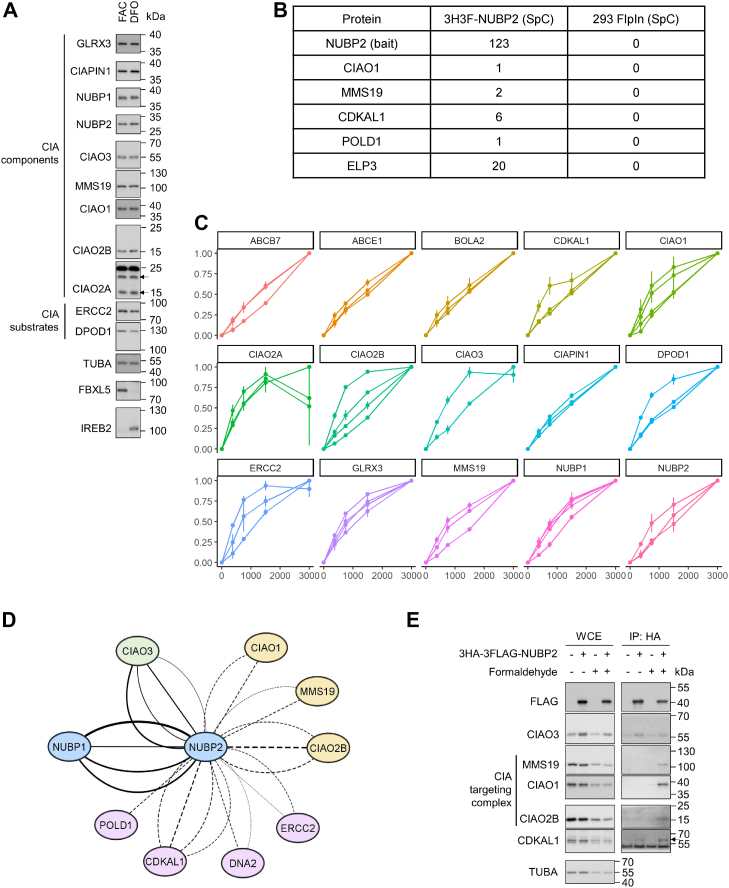
**CIA components and substrates form higher order complexes**. *A*, HEK293 cells were treated with 100 μg/ml ammonium ferric citrate (FAC) or 100 μM deferoxamine (DFO) for 8 h to create an iron-sufficient or -deficient environment, respectively. Whole cell extracts (WCEs) were resolved by SDS-PAGE and blotted with antibodies against known components of the cytosolic iron–sulfur cluster assembly (CIA) pathway, CIA substrates, loading control α-tubulin, FAC treatment control FBXL5, and DFO treatment control IREB2. *B*, Flp-In T-Rex 293 cell line (Flp-In 293) engineered to stably express NUBP2 was induced overnight with 1 μg/ml doxycycline. Affinity purified NUBP2 and associated proteins were identified by bottom-up proteomics. Data-dependent acquisition of two technical replicates was performed. Spectra count (SpC) for selected proteins combined from the two technical replicates was shown. *C*, parallel reaction monitoring was conducted on HEK293 whole cell lysate with indicated amounts of protein using a targeted proteomics assay that monitors the abundance of known CIA factors (ABCB7, GLRX3, BOLA2, CIAPIN1, NUBP1, NUBP2, CIAO3, CIAO1, CIAO2B, CIAO2A, MMS19) and prototypical substrates (CDKAL1, DNA2, ERCC2, POLD1, and ABCE1). Two technical replicates were acquired. Intensities were normalized for each precursor to the highest intensity in a replicate. *D*, Flp-In 293 cells that stably express NUBP2 were induced with 1 μg/ml doxycycline. Affinity purified NUBP2 and associated proteins were identified by acquisition of a targeted proteomic assay containing CIA scaffold complex components (NUBP1 and NUBP2), CIAO3, CIA targeting complex components (MMS19, CIAO1, and CIAO2B) and prototypical substrates (CDKAL1, DNA2, ERCC2, POLD1, and ABCE1). Two biological replicates were performed. Acquired spectra were searched with MaxQuant. Each edge represents a peptide identified. Solid edges connect bait protein to known interactors while dashed edges connect to novel interactors discovered in our study. Edge widths correspond to the posterior error probability of each peptide. *E*, Flp-In 293 background cells or cells expressing NUBP2 were either directly harvested or after treatment with 1% formaldehyde. Anti-HA immunoprecipitation were performed. WCEs and anti-HA immunoprecipitates were blotted for CIAO3, CIA targeting complex components, and the CIA substrate CDKAL1. CIAO1: probable cytosolic iron-sulfur protein assembly protein CIAO1; CIAO2B: cytosolic iron-sulfur assembly component 2B; CIAO3: cytosolic iron-sulfur assembly component 3; MMS19: MMS19 nucleotide excision repair protein homolog; NUBP1: nucleotide-binding protein 1; NUBP2: nucleotide-binding protein 2.

In order to investigate how the CIA pathway responds to changes in cellular environment, we began by comparing the endogenous protein levels of major CIA components in cells exposed to iron supplementation or chelation, mimicking an iron sufficient or deficient environment. Cells were treated with ferric ammonium citrate (FAC) or the iron chelator deferoxamine mesylate (DFO) for 8 h, and whole cell lysates were probed with antibodies against CIA components as well as two known substrates. Protein levels for F-box/LRR-repeat protein 5 (FBXL5), an E3 ligase that accumulates when sufficient iron is present, and iron-responsive element-binding protein 2 (IREB2/IRP2), an FBXL5 substrate that is stabilized by iron depletion, served as treatment controls for FAC and DFO, respectively (20). We did not observe significant changes in the steady-state levels of either CIA components or CIA substrates in this time frame (Fig. 1*A*). Although defects in Fe-S cluster incorporation have been previously shown to cause destabilization of a number of Fe-S proteins, these effects are typically observed either as a result of chronic ablation of the Fe-S cluster assembly machinery or in mutant proteins defective in cluster incorporation (2, 21).

Since we did not observe any immediate effect of changes in intracellular iron levels on the stability of CIA factors and substrates, we next examined interactions between CIA components under basal conditions. Multiple independent studies have shown that components of the CIA targeting complex are detected in higher order complexes ranging in molecular weight from 400 to 1000 kDa (16, 22, 23). Components of the CIA targeting complex interact with CIAO3, which in turn interacts with the CIA scaffold complex, indicating that these CIA components may be organized into higher order complexes. To examine this possibility, we performed affinity purification of NUBP2, a component of the CIA scaffold complex, and characterized proteins associated with NUBP2 using an unbiased shotgun proteomic approach. In addition to NUBP1 and CIAO3 which are known NUBP2 interactors, we also detected two components of the CIA targeting complex (CIAO1 and MMS19) as well as CIA substrates (CDKAL1 and ELP3) in the NUBP2 immunoprecipitates (Fig. 1*B* and Table S1). CIAO2B, another component of the targeting complex, was not identified in this analysis, although this could be due to poor sampling of low abundance peptides. To address this potential issue, we developed a targeted proteomics assay (tier 3) that utilizes parallel reaction monitoring (PRM) to assess the presence and abundance of a panel of proteins relevant to the Fe-S cluster assembly pathways (24). We first tested this assay on a serially diluted peptide standard prepared from HEK293 whole cell extracts and were able to detect the presence and estimate the relative abundance of known CIA factors (ABCB7, BOLA2, GLRX3, CIAPIN1, NUBP1, NUBP2, CIAO3, MMS19, CIAO1, CIAO2B, and CIAO2A) and several prototypical CIA substrates (ABCE1, CDKAL1, ERCC2, and POLD1) (Fig. 1*C*). This targeted approach, which provides better sensitivity and quantitation than unbiased proteomics assays, was then applied to NUBP2 immunoprecipitates to specifically monitor diagnostic peptides derived from components of the CIA scaffold complex (NUBP1 and NUBP2), CIAO3, components of the CIA targeting complex (MMS19, CIAO1, and CIAO2B), and CIA substrates (CDKAL1, DNA2, ERCC2, ABCE1, and POLD1). Diagnostic peptides utilized in this analysis are listed in Table S2. As expected, NUBP1 and CIAO3 were identified as interacting proteins (Fig. 1*D* and Table S3). All components of the CIA targeting complex (MMS19, CIAO1, and CIAO2B) were also found associated with NUBP2. In addition, we observed that CIA substrates CDKAL1, DNA2, ERCC2, and POLD1 copurified with NUBP2 (Fig. 1*D*). These observations together suggest that the CIA scaffold complex, CIAO3, the CIA targeting complex, and CIA substrates potentially assemble into a higher order protein assembly that facilitates Fe-S cluster transfer into substrates. The assembly is likely dynamic, given that formaldehyde crosslinking enhanced the association between the CIA scaffold complex and the CIA targeting complex (Fig. 1*E*).

### Iron regulation of CIAO3 interactome

**Figure 2:**
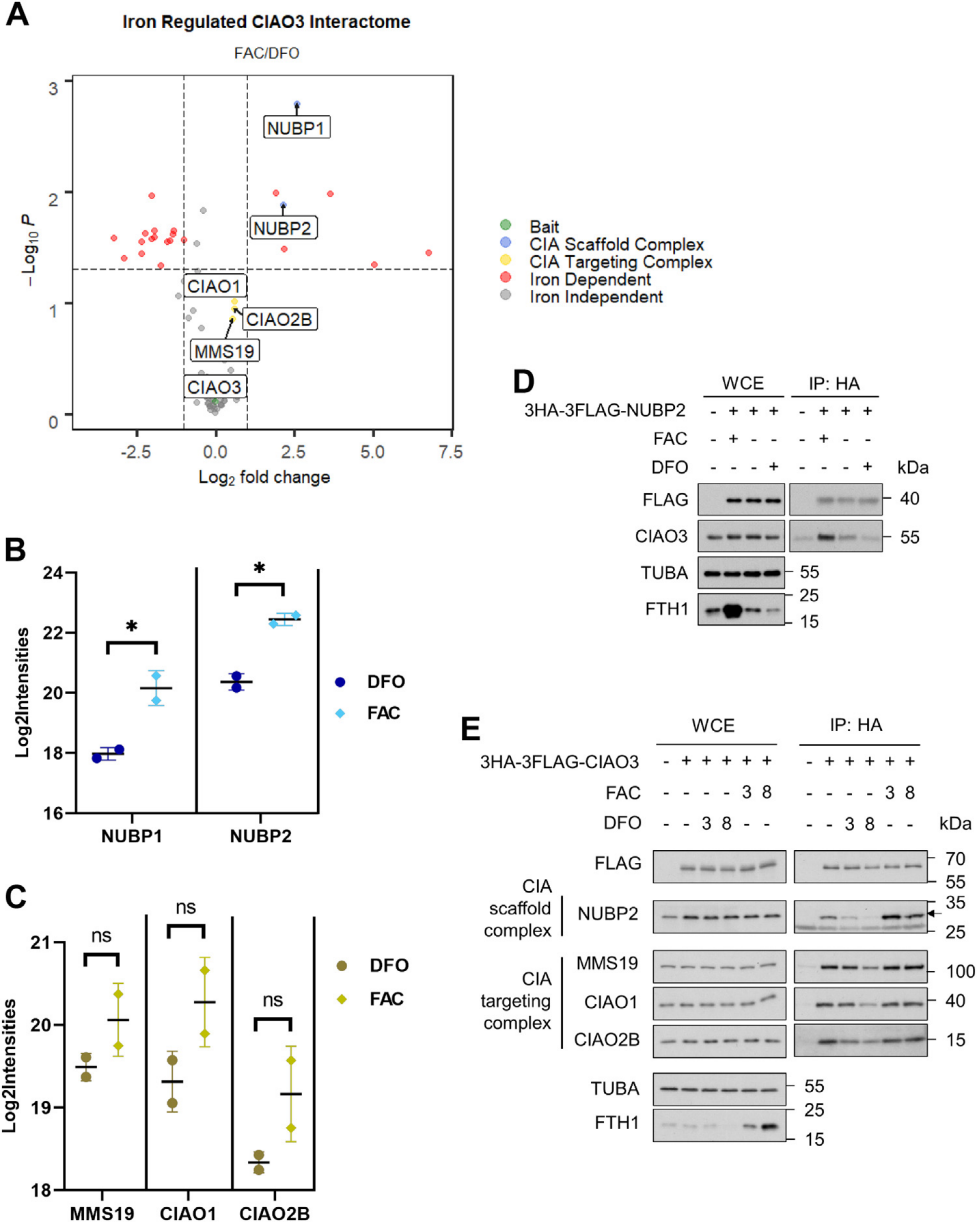
**CIAO3 interactions are regulated by intracellular iron level in a time-dependent manner**. *A–C*, Flp-In 293 stably expressing 3HA-3FLAG–tagged CIAO3 was induced with 1 μg/ml doxycycline for 24 h and treated with 100 μg/ml FAC or 100 μM DFO for 8 h before harvesting. Two biological replicates were performed for each experiment. *A*, affinity purified CIAO3 and associated proteins were characterized by shotgun proteomics and quantified by MS1 intensity-based label-free quantification. Abundance of co-eluted (*B*) CIA scaffold complex components (NUBP1 and NUBP2) and (*C*) CIA targeting complex components (MMS19, CIAO1, and CIAO2B) were monitored using a targeted proteomic assay. Log2 of calculated protein intensities (Log2Intensities) under FAC or DFO condition were plotted with mean ± SD. ∗ denotes *p* < 0.05. *D*, Flp-In 293 cells or Flp-In 293 expressing NUBP2 were induced with 1 μg/ml doxycycline for 24 h. Cells expressing 3HA-3FLAG-NUBP2 were treated with 100 μg/ml FAC, untreated or treated with 100 μM DFO for 8 h before harvesting. WCEs and anti-HA immunoprecipitates (IP: HA) were analyzed by immunoblotting with antibodies against FLAG, CIAO3, loading control α-tubulin, and FAC/DFO treatment control FTH1. *E*, Flp-In 293 background cells or cells stably expressing 3HA-3FLAG-CIAO3 were induced with 1 μg/ml doxycycline overnight, untreated, or treated with 100 μg/ml FAC or 100 μM DFO for hours indicated. WCEs and anti-HA immunoprecipitates (IP: HA) were blotted with antibodies against indicated proteins. CIAO3, cytosolic iron–sulfur assembly component 3; CIAO1: probable cytosolic iron-sulfur protein assembly protein CIAO1; CIAO2B: cytosolic iron-sulfur assembly component 2B; CIAO3: cytosolic iron-sulfur assembly component 3; DFO, deferoxamine; FAC, ferric ammonium citrate; MMS19: MMS19 nucleotide excision repair protein homolog; NUBP1: nucleotide-binding protein 1; NUBP2: nucleotide-binding protein 2; WCE, whole cell extract.

To determine the influence of intracellular iron availability on the assembly of CIA complexes, we utilized affinity purification of CIAO3 followed by tandem mass spectrometry to compare the CIAO3 interactome between iron-replete and iron-depleted conditions. These proteomics studies were done using both standard unbiased protein identification followed by label-free quantitation as well as our targeted proteomics assay that specifically measures the abundance of a panel of key Fe-S machinery proteins and substrates. The unbiased proteomics analysis demonstrated that interactions between CIAO3 and multiple proteins depend on the availability of labile iron, including both components of the CIA scaffold complex (NUBP1 and NUBP2) (Fig. 2*A* and Table S4). In contrast, interactions between CIAO3 and the CIA targeting complex were minimally affected by cellular iron levels (Fig. 2*A*). These results were validated by our targeted proteomics assay in which PRM was used to detect and quantify the levels of the CIA scaffold complex and the CIA targeting complex present in CIAO3 immunoprecipitates isolated from iron-replete and iron-depleted conditions. We confirmed that CIAO3 interacts with NUBP1 and NUBP2 in an iron-dependent manner and observed that both CIAO3–NUBP1 and CIAO3–NUBP2 interactions were reduced ∼4-fold in iron-deficient conditions indicating that CIAO3 dissociates from the intact CIA scaffold complex (Fig. 2*B*). Conversely, interactions between CIAO3 and the CIA targeting complex were only subtly influenced by iron levels and did not reach statistical significance (Fig. 2*C*).

To validate that endogenous CIAO3 also interacts with the CIA scaffold complex in an iron-dependent manner, we treated cells expressing 3HA-3FLAG–tagged NUBP2 with FAC or deferoxamine mesylate, immunoprecipitated NUBP2 from whole cell lysate using anti-HA beads, and immunoblotted with CIAO3 antibodies. Our data show that the CIAO3–NUBP2 interaction is stabilized by the addition of iron and impaired when iron is depleted through chelation (Fig. 2*D*).

We further characterized the association between CIAO3 and its interactors in response to alterations in intracellular iron levels after different time periods of treatment. We treated cells expressing 3HA-3FLAG-CIAO3 with FAC or DFO for either 3 or 8 h. We performed anti-HA immunoprecipitation followed by immunoblotting with indicated antibodies (Fig. 2*E*). Our data showed that the CIAO3–NUBP2 interaction increased after 3 h of FAC treatment but slightly declined by 8 h of treatment. Iron chelation caused a strong reduction in NUBP2 binding to CIAO3 as early as 3 h after treatment with DFO and extended up to at least 8 h after treatment. Ferritin levels, as expected, were gradually increasing over the same time period. These observations suggest that the response of the CIAO3–NUBP2 interaction to changes in iron levels is rapid.

### CIAO3 interactions are redox regulated

**Figure 3:**
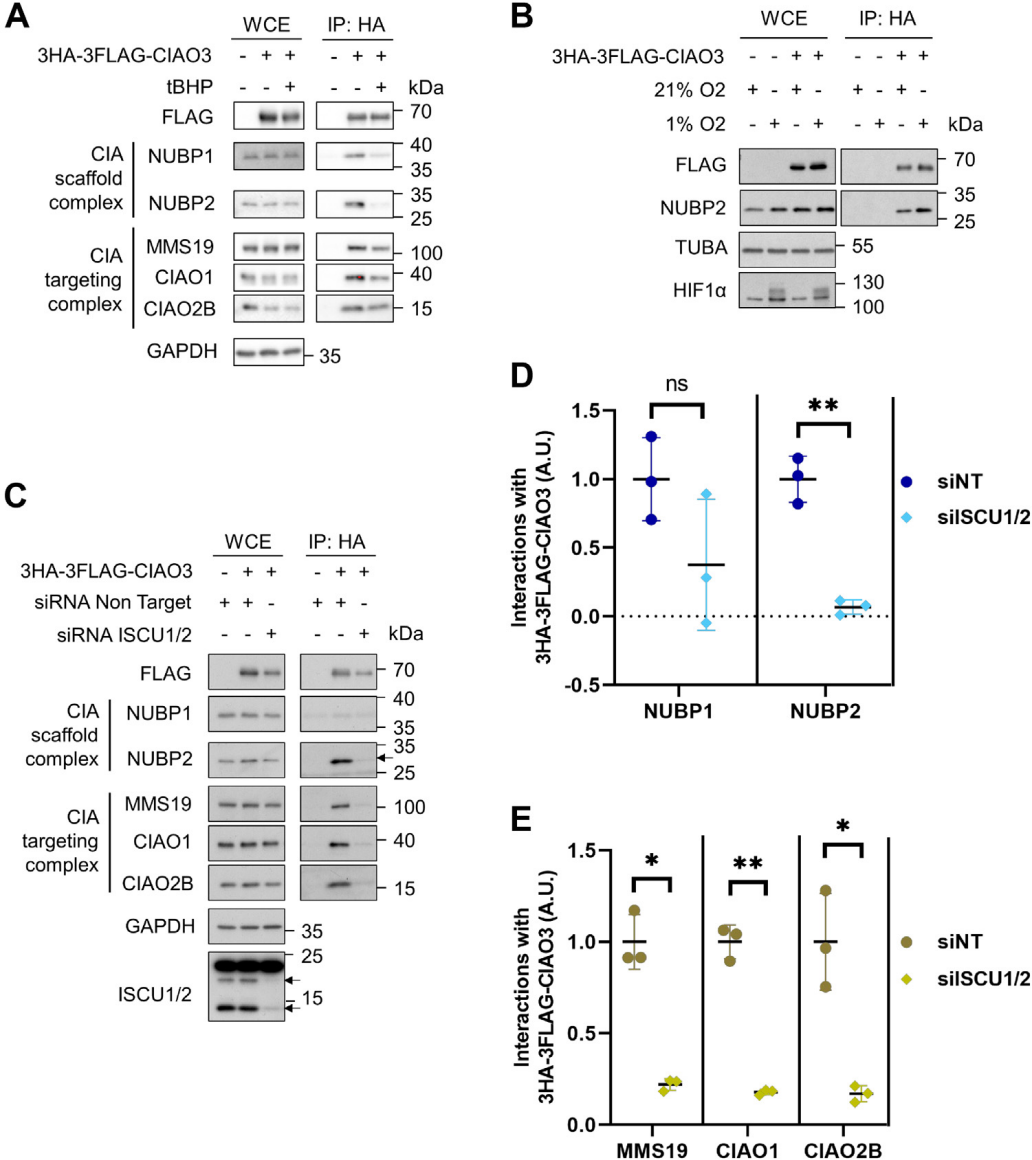
**CIAO3 interactions are altered by changes in cellular redox state and requires functional mitochondrial Fe-S cluster biogenesis**. *A*, Flp-In 293 cells expressing 3HA-3FLAG-CIAO3 were treated with tert-butyl hydroperoxide (tBHP) for 4 h to introduce reactive oxygen species. WCEs and anti-HA immunoprecipitates were blotted with antibodies against indicated proteins and loading control GAPDH. *B*, Flp-In 293 cells expressing 3HA-3FLAG-CIAO3 or control cells were induced with 1 μg/ml doxycycline overnight and cultured in 21% O_2_ or 1% O_2_ for 16 h before harvesting. WCEs and anti-HA immunoprecipitates were immunoblotted with antibodies against NUBP2, loading control α-tubulin, and hypoxia treatment control HIF1α. *C*, mitochondrial iron-sulfur cluster biogenesis was disrupted by using siRNA to silence ISCU1/2 in Flp-In 293 cells for 48 h while control cells were treated with nontarget siRNA. Doxycycline was added to induce expression of 3HA-3FLAG-CIAO3. WCEs and HA immunoprecipitates were blotted with indicated antibodies. *D* and *E*, quantification of (*C*) by densitometry. Protein abundance of coimmunoprecipitated proteins were normalized to the protein level of immunoprecipitated bait (3HA-3FLAG-CIAO3). Mean ± SD was plotted for n = 3 independent experiments. ∗*p* < 0.05, ∗∗*p* < 0.01. CIAO3, cytosolic iron–sulfur assembly component 3; CIAO1: probable cytosolic iron-sulfur protein assembly protein CIAO1; CIAO2B: cytosolic iron-sulfur assembly component 2B; CIAO3: cytosolic iron-sulfur assembly component 3; Fe-S, iron–sulfur; MMS19: MMS19 nucleotide excision repair protein homolog; NUBP1: nucleotide-binding protein 1; NUBP2: nucleotide-binding protein 2; WCE, whole cell extract.

Given the observation that CIAO3’s interactome was regulated by iron availability, we next examined whether these interactions were also influenced by other environmental stimuli. First, we treated cultured cells with ROS and examined the effects on the CIAO3 interactome. Tert-butyl hydroperoxide (tBHP) oxidizes glutathione and induces oxidative stress (25). After cells were exposed to tBHP for 4 h, 3HA-3FLAG-tagged CIAO3 was immunoprecipitated from whole cell extracts and immunoblotted with antibodies against both the CIA scaffold complex and the CIA targeting complex. We observed a diminished CIAO3 interaction with the CIA scaffold complex under oxidative stress (Fig. 3*A*). A decreased interaction between CIAO3 and the CIA targeting complex was also observed but was comparatively modest under the same conditions. In addition to oxidative stress, we also manipulated oxygen tension and examined its effect on CIAO3 interactions. We immunoprecipitated 3HA-3FLAG-CIAO3 from extracts derived from cells cultured in either 21% O_2_ or 1% O_2_ and determined its interactions by immunoblotting with antibodies of NUBP2. HIF1α served as a positive control for hypoxia. We found that the CIAO3–NUBP2 interaction was stabilized in cells cultured in 1% O_2_ (Fig. 3*B*). Together these results suggest that CIAO3-containing complexes are sensitive to the redox status of the cell.

The versatile nature of Fe-S clusters allows them to sense changes in both intracellular iron availability and the redox status of the cell (1, 26). As such, we hypothesized that the ability of these environmental changes to influence CIAO3 interactions might stem from effects on the Fe-S clusters bound to CIAO3 or other key components of this pathway. To test this possibility, we examined how disruption of Fe-S cluster biogenesis pathways affected the CIAO3 interactome. Previous studies have shown that cytosolic Fe-S cluster biogenesis mediated by the CIA pathway depends on mitochondrial Fe-S cluster biogenesis by the ISC pathway and that depleting the ISC scaffold protein, ISCU, leads to reduced iron incorporation and protein stability of both CIAO3 and CIA substrates (2, 27). We depleted ISCU1/2 from cells using RNAi and then induced expression of 3HA-3FLAG-CIAO3 which was stably expressed under the control of a doxycycline-inducible promoter. We observed a reduced amount of CIAO3 in the cells with silenced ISCU1/2, which is consistent with previous observations (Fig. 3*C*) (2). Immunoblots of the affinity-purified CIAO3 complexes showed reduced co-precipitation for both the CIA scaffold complex and the CIA targeting complex. Densitometric evaluation of CIAO3 interactions as shown in Figure 3*C* revealed >60% and >90% reduction in the amount of NUBP1 and NUBP2 co-purifying with CIAO3 in response to the silencing of ISCU1/2 (Fig. 3*D*). In addition, we observed that the interactions between CIAO3 and components of the CIA targeting complex also drastically diminished upon knockdown of ISCU1/2 (Fig. 3*E*). Together, our observations demonstrate the assembly of CIAO3 into higher order complexes depends on the presence of a functional Fe-S cluster biogenesis pathway.

### Fe-S cluster incorporation of CIAO3 regulates its interactions

**Figure 4:**
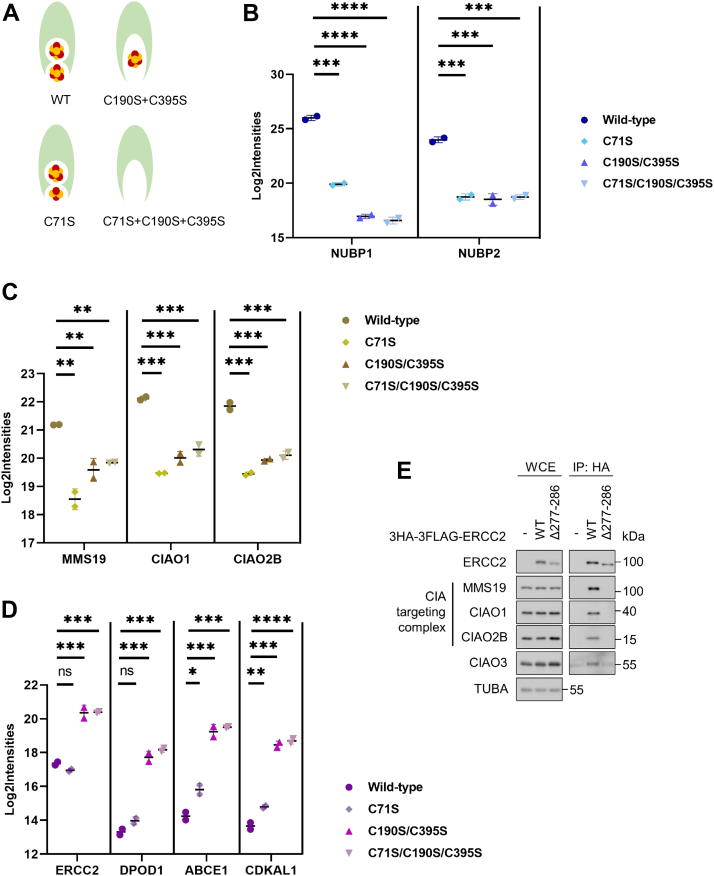
**Fe-S cluster incorporation into CIAO3 controls its interactions.***A*, schematic representation of Fe-S incorporation in wildtype CIAO3 and mutants. *B*–*D*, Flp-In 293 cells that stably express wildtype or mutant CIAO3 were induced with 1 μg/ml doxycycline for 24 h before harvesting. CIAO3 and associated proteins were affinity purified. Two biological replicates were analyzed. Protein abundances were monitored by a targeted proteomic assay containing known CIA components and a subset of prototypical CIA substrates after normalization to CIAO3 levels. Log2 transformed abundance of the co-eluted proteins (Log2Intensiteis) were plotted for the CIA scaffold complex (*B*), the CIA targeting complex (*C*), and selected CIA substrates (*D*). Mean ± SD was indicated. Protein levels of interactors co-eluted with mutant CIAO3 were compared to wildtype. ∗*p* < 0.05, ∗∗*p* < 0.01, ∗∗∗*p* < 0.001 and ∗∗∗∗*p* < 0.0001. *E*, Flp-In 293 control cells, cells expressing wildtype 3HA-3FLAG-ERCC2 and cells expressing mutant ERCC2 lacking the MMS19 binding region were induced with 1 μg/ml doxycycline for 24 h. Cell lysates were immunoprecipitated. WCEs and HA immunoprecipitates were blotted with indicated antibodies. CIA, cytosolic iron–sulfur cluster assembly; CIAO3, cytosolic iron–sulfur assembly component 3; CIAO1: probable cytosolic iron-sulfur protein assembly protein CIAO1; CIAO2B: cytosolic iron-sulfur assembly component 2B; CIAO3: cytosolic iron-sulfur assembly component 3; Fe-S, iron–sulfur; MMS19: MMS19 nucleotide excision repair protein homolog; NUBP1: nucleotide-binding protein 1; NUBP2: nucleotide-binding protein 2; WCE, whole cell extract.

CIAO3, which plays an essential role in bridging early and late CIA steps, has two Fe-S cluster binding sites: one at its N terminus and the other at its C terminus (28, 29). Given that Fe-S clusters are intrinsically sensitive to the cellular environment and regulate the stability and/or function of Fe-S proteins, we reasoned that CIAO3 interactions may be regulated by its cluster incorporation status. Previous studies have indicated that missense mutations of CIAO3 substituting cysteine with serine at position 71 in the N terminus or at both positions 190 and 395 in the C terminus render the protein defective in binding of Fe-S clusters (28, 29). Based on these studies, we generated CIAO3 mutants with impaired cluster incorporation (C71S, C190S/C395S, and C71S/C190S/C395S) to determine whether the Fe-S cluster requirement observed for CIAO3 interactions was dependent on cluster binding by CIAO3 itself (Fig. 4*A*). To compare and quantify the interactomes of wildtype and mutant versions of CIAO3 with known CIA components, we utilized our PRM-based targeted proteomics assay that monitors known CIA components and substrates as described earlier. We purified both wildtype and mutant 3HA-3FLAG CIAO3 complexes and quantified their interactions with the CIA scaffold complex, the CIA targeting complex, and CIA substrates after normalization to the amount of CIAO3 present in each purification. We observed that CIAO3-NUBP1/2 interactions were dramatically reduced for more than 32-fold, consistent with our earlier observation that Fe-S clusters are required for the interaction between CIAO3 and the CIA scaffold complex (Fig. 4*B*). The CIA targeting complex also showed modestly reduced association with CIAO3 displaying an approximately 5-fold decrease for the C71S mutant, a 3- to 4-fold decrease for the C190S/C395S mutant, and a 2-fold change in the C71S/C190S/C395S mutant (Fig. 4*C*). Intriguingly, we observed that the association of substrates like ABCE1 and CDKAL1 with CIAO3 strongly increased when the CIAO3 C-terminal Fe-S cluster binding site was mutated (Fig. 4*D*), which suggests that CIAO3 may also associate with CIA substrates independently of the CIA targeting complex. Of note, we previously generated a mutant of ERCC2 lacking amino acids 277 to 286 that cannot bind to the CIA targeting complex (18). We show here that this ERCC2 mutant associated more weakly with CIAO3 relative to wildtype ERCC2, suggesting that CIAO3 binding by ERCC2 requires the CIA targeting complex binding region of ERCC2 and is consistent with the model that the CIA scaffold complex, CIAO3, the CIA targeting complex, and CIA substrates form higher order complexes that facilitates Fe-S protein maturation (Fig. 4*E*). These results together provide evidence that Fe-S cluster incorporation into CIAO3 controls its interactions and governs its incorporation in CIA metabolon.

### Disease-associated CIAO3 mutant fails to assemble into CIA metabolon

**Figure 5:**
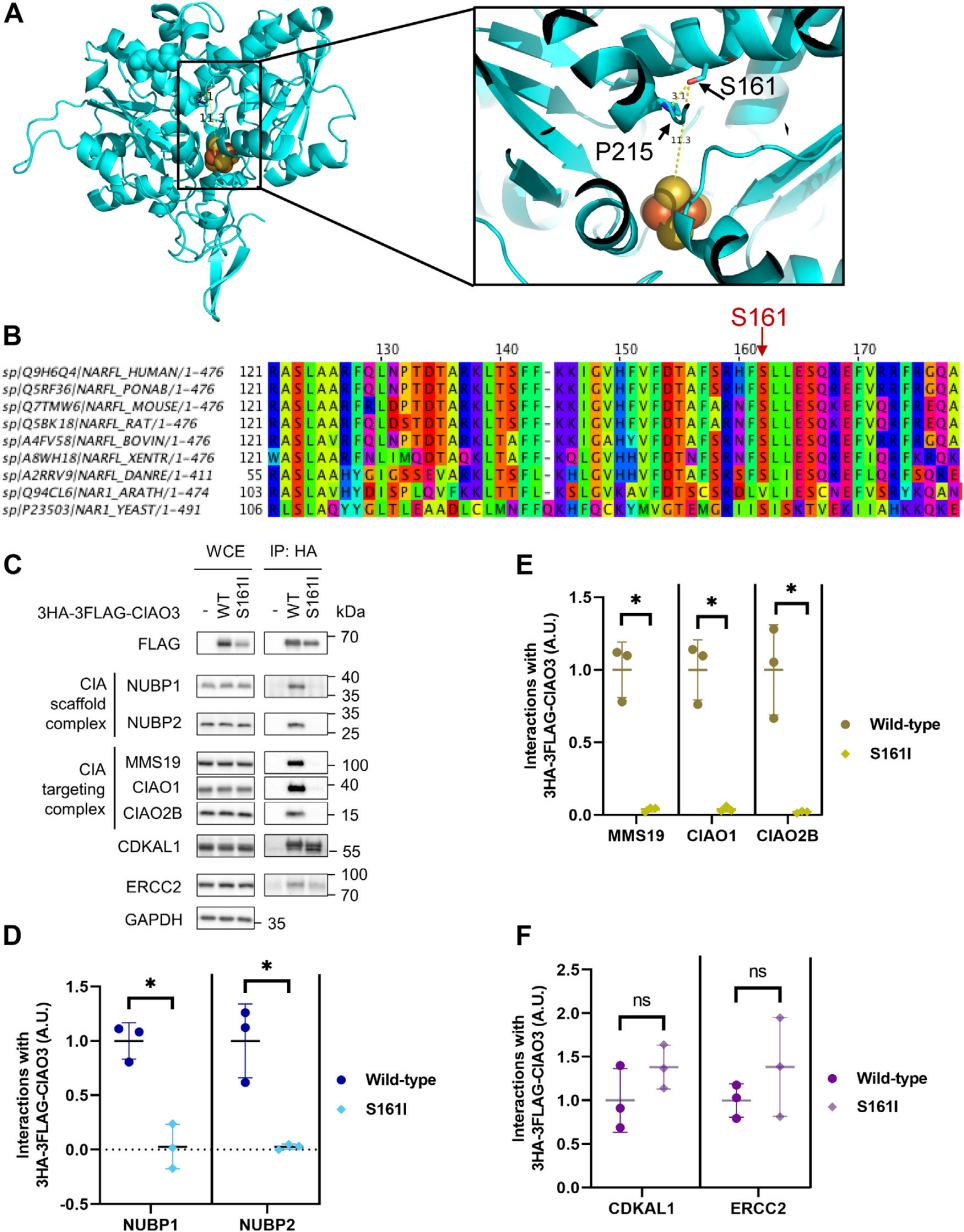
**CIAO3 mutant associated with PAVMs cannot assemble into the higher order complexes**. *A*, homology modeled human CIAO3 from iron-only hydrogenase (1FEH) showing distances from serine 161 to proline or to the C-terminal Fe-S cluster. *B*, sequence alignment of CIAO3 orthologs. *C*, WCEs and HA immunoprecipitates from Flp-In 293 cells expressing wildtype CIAO3, the S161I mutant, or control cells were analyzed by SDS-PAGE and immunoblotted with antibodies indicated. *D*–*F*, quantification of (*C*) by densitometry showing baseline corrected protein abundance of coimmunoprecipitated proteins with respect to the bait protein (3HA-3FLAG-CIAO3 wild-type or S161I). Mean ± SD was plotted for n = 3 independent experiments. ∗*p* < 0.05. CIAO3, cytosolic iron–sulfur assembly component 3; CIAO1: probable cytosolic iron-sulfur protein assembly protein CIAO1; CIAO2B: cytosolic iron-sulfur assembly component 2B; CIAO3: cytosolic iron-sulfur assembly component 3; MMS19: MMS19 nucleotide excision repair protein homolog; NUBP1: nucleotide-binding protein 1; NUBP2: nucleotide-binding protein 2; PAVMS, pulmonary arteriovenous malformations; WCE, whole cell extract; Fe-S, iron–sulfur.

A mutation in CIAO3 (S161I) has recently been reported to associate with diffuse pulmonary arteriovenous malformations (PAVMs) (30). Homology modeling of human CIAO3 based on Fe-only hydrogenase of *Clostridium pasteurianum* (1FEH) revealed that this evolutionally conserved serine 161 is ∼3.1 Å from an evolutionally conserved proline (P215) and 11.3 Å from the C-terminus Fe-S cluster (Fig. 5, *A* and *B*) (31). Therefore, we hypothesized that the CIAO3-S161I mutation might perturb Fe-S cluster binding due to either the loss of serine–proline hydrogen bonding or sterically hindering cluster binding in the C-terminus site. In this case, the CIAO3-S161I would behave phenotypically like the C-terminal site CIAO3 mutants (C190S/C395S and C71S/C190S/C395S) with decreased binding to the CIA scaffold complex and the CIA targeting complex but increased binding to CIA substrates. To test this, we immunoprecipitated both wildtype and S161I versions of 3HA-3FLAG-CIAO3 and probed the immunoprecipitates for components of the CIA scaffold complex (NUBP1 and NUBP2), components of the CIA targeting complex (MMS19, CIAO1, and CIAO2B), and CIA substrates (CDKAL1 and ERCC2) (Fig. 5*C*). Relative to wildtype CIAO3, the interactions of CIAO3-S161I with both the CIA scaffold complex and the CIA targeting complex were significantly reduced (Fig. 5, *D* and *E*), while its association with CIA substrates increased (Fig. 5*F*), reminiscent of C190S/C395S and C71S/C190S/C395S mutants. These findings suggest that the failure of CIAO3-S161I to incorporate into a higher order complex may contribute to the disease phenotype associated with PAVMs.

## Discussion

**Figure 6:**
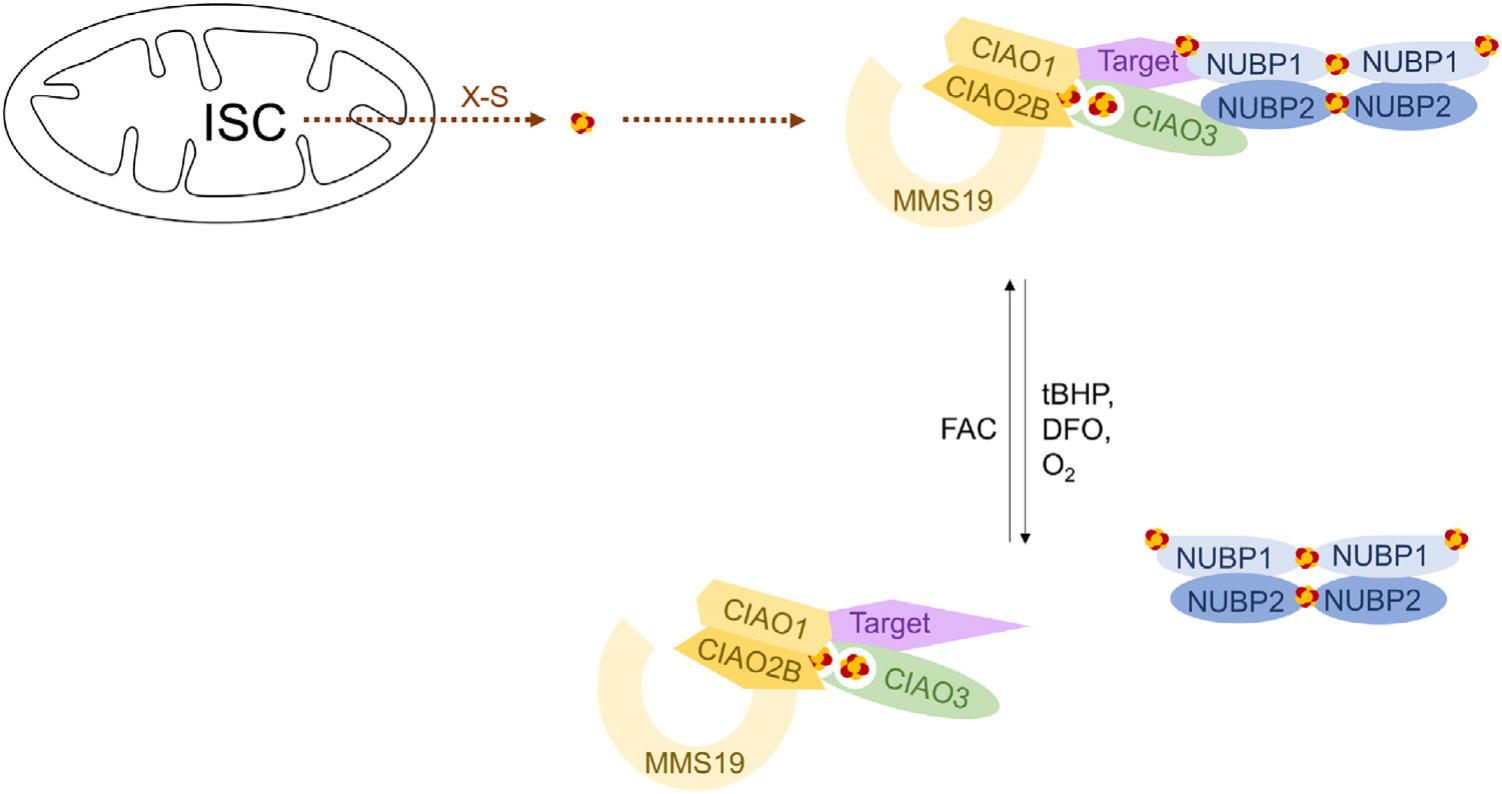
**Model for the regulated assembly of the CIA machinery in response to ROS, O**_**2**_**tension, intracellular iron levels, and Fe-S cluster assembly on CIAO3**. CIA, cytosolic iron–sulfur cluster assembly; CIAO3, cytosolic iron–sulfur assembly component 3; CIAO1: probable cytosolic iron-sulfur protein assembly protein CIAO1; CIAO2B: cytosolic iron-sulfur assembly component 2B; CIAO3: cytosolic iron-sulfur assembly component 3; Fe-S, iron–sulfur; MMS19: MMS19 nucleotide excision repair protein homolog; NUBP1: nucleotide-binding protein 1; NUBP2: nucleotide-binding protein 2; ROS: reactive oxygen species.

The CIA pathway facilitates Fe-S cluster incorporation into a plethora of extramitochondrial Fe-S proteins involved in a variety of essential cellular functions. It remains a key question how the CIA pathway adapts to different cellular environments to achieve precise control of substrate maturation. In this study, we describe a novel axis of regulation for cytosolic [4Fe-4S] cluster biogenesis (Fig. 6). Utilizing a targeted proteomics assay to assess known components and substrates of the CIA pathway, we demonstrate the existence of higher order CIA complexes containing the CIA scaffold complex, CIAO3, the CIA targeting complex, and CIA substrates. These higher order complexes are sensitive to acute environmental changes and are reorganized in response to changes in the labile iron pool, oxygen tension, and ROS. Our data further show that Fe-S cluster binding by CIAO3 is required for its interactions with the CIA scaffold complex and the CIA targeting complex. Finally, we demonstrate that the CIAO3-S161I mutant associated with diffuse pulmonary arteriovenous malformation fails to incorporate into a functional CIA complex highlighting the physiological and pathological relevance of this pathway.

Although an understanding of the components and the organization of the CIA pathway has begun to emerge, still very little is known about the dynamics of the pathway and how it responds to different cellular and environmental cues, especially in the mammalian system. A previous study has demonstrated that the association of BOLA2 with GLRX3 in the context of the cytosolic [2Fe-2S] cluster biogenesis machinery is iron dependent and highlights one important mode of regulation (17). Our study further extends this paradigm by showing that the association of CIAO3 with the CIA scaffold complex is tightly coupled to cellular iron levels with iron strongly promoting the assembly. The regulated binding of CIAO3 with the CIA scaffold complex is also influenced by ROS and hypoxia suggesting that it is broadly responsive to changes in cellular conditions. These data suggest that the CIA pathway adapts to acute environmental cues through the reorganization of a higher order CIA complex *via* a mechanism by which the CIA scaffold complex dynamically joins/leaves the rest of the complex.

Unlike acute environmental changes that primarily alter CIAO3’s association with the CIA scaffold complex, compromised Fe-S cluster biogenesis and improper cluster incorporation in CIAO3 prohibited both the CIA scaffold complex and the CIA targeting complex from interacting with CIAO3. We therefore reasoned that the two Fe-S clusters in CIAO3, directly or indirectly, sense the changes in the cellular environment and subsequently regulate the dynamic assembly of the CIA machinery. Homology structural modeling predicts that the N-terminus Fe-S cluster of CIAO3 is solvent exposed while the C-terminus cluster is buried in the center of the protein. We speculate that these clusters play different roles in regulating CIAO3 behavior with the solvent exposed N-terminal Fe-S cluster responding to acute changes in the cellular environment to modulate CIAO3 interactions and the C-terminal cluster being required for the structural integrity of the protein but having limited capacity for immediate environmental sensing due to its solvent inaccessibility. The CIAO3-S161I mutant was found to associate with diffuse PAVMs (30). Based on our predicted structure of CIAO3, substituting Ser with Ile at residue 161 would disrupt the C-terminal integrity of protein and likely phenocopy mutants with defective C-terminal Fe-S cluster incorporation. As expected, we observed a reduction in protein levels for the CIAO3-S161I mutant. This mutant also interacts weakly with the CIA scaffold complex and the CIA targeting complex. These findings are consistent with the model that the C-terminus cluster of CIAO3 mediates its stability and that Fe-S cluster binding in CIAO3 is required for its incorporation into CIA machineries. The CIAO3-S161I mutation in patients likely disrupts cytosolic Fe-S cluster biogenesis and potentially contributes to the molecular basis of PAVMs.

## Experimental procedures

### Plasmids and primers

Plasmid containing wildtype human CIAO3 was purchased from Open Biosystems (Clone: 5242707). cDNA was amplified using Phusion polymerase with the primer pair containing attB recombination sites (5′-GGGGACAAGTTTGTACAAAAA AGCAGGCTTCATGGCGTCGCCCTTCAGC-3′; 5′- GGGG ACCACTTTGTACAAGAAAGCTGGGTCCTACCACCGGA TGCCCAG-3′). Using the Gateway Recombination Cloning Technology, CIAO3 was cloned into pDONR221 vector and subsequently into the destination vector pcDNA5/FRT encoding a N-terminus tandem 3xHA-3xFLAG tag. CIAO3-C71S, CIAO3-C190S/C395S, CIAO3-C71S/C190S/C395S, and CIAO3-S161I were generated using Quikchange Mutagenesis (Agilent) with primer pairs containing the mutated nucleotides (C71S: 5′-CTAAACGACTCCCTGGCGTGC-3′, 5′-GCACGCCAGGGAGTCGTTTAG-3′; C190S: 5′-GCCTC TGCCTCCCCAGGCTGG-3′, 5′-CCAGCCTGGGGAGGCAGAGGC-3′; C395S: 5′-GTCATGGCCTCCCCCTCAGGC-3′, 5′- GCCTGAGGGGGAGGCCATGAC-3’; S161I: 5′-CTCTC CAGGAGGATGAAGTGCCTTGAGAAGGC-3′, 5′-GCCTT CTCAAGGCACTTCATCCTCCTGGAGAG-3′). Mutations were verified by sequencing with M13F and M13R primers.

### Cell culture, cell lines, transfection, and treatments

HEK293 cells were obtained from the American Type Culture Collection. The Flp-In T-REx 293 cell line obtained from Thermo Fisher Scientific was used to generate HEK293 cells stably expressing 3xHA-3xFLAG-tagged wildtype and mutant CIAO3 using the Flp-In System. Cells were cultured in Dulbecco’s Modified Eagle’s Medium (Gibco 11960-044) supplemented with 10% fetal bovine serum (Gemini Bio-products Foundation B 900-208), 2 mM L-Glutamine (Gibco 25030-081), with or without 1× Antibiotic-Antimycotic (Gibco 15240-062) at 37 °C. Cells grown under normoxic conditions were cultured in ambient air with 5% CO_2_. Hypoxic conditions were maintained by culturing cells for 16 h in a hypoxia chamber (STEMCELL Technologies) equilibrated with a gas mixture containing 1% O_2_, 5% CO_2_ and 94% N_2_ at a flow rate of 20 L/min for 7 min using the Single Flow Meter (STEMCELL Technologies, Cat #27311) and then sealed till harvesting. The Flp-In T-REx 293 cell lines were treated with 1 μg/ml doxycycline (Fisher Bioreagents #BP26535) for about 24 h to induce protein expression. Cells were treated with drugs including 100 μg/ml FAC (Fisher Bioreagents CAS 1185-57-5), 100 μM DFO (Sigma D9533-1G), or 100 μM tBHP (Aldrich 458139-100 ML, Lot # MKCD3313). Knockdown of ISCU1/2 was achieved by transfecting cells with siGENOME Human ISCU siRNA (Dharmacon 15240–062 SMARTPool M-012837-03-0005) using the Lipofectamine RNAiMAX transfection reagent and the manufacturer’s protocol.

### Co-immunoprecipitation and immunoblotting analysis

Cell pellets were resuspended in lysis buffer (100 mM Tris-HCl pH 8.0, 150 mM NaCl, 5% glycerol, 0.1% NP-40, 1 mM DTT, 1 mM AEBSF, 1 μg/ml or 10 μM leupeptin, 1 μM pepstatin A and 1× phosphatase inhibitor). Lysates were cleared by centrifugation at 13,200 RPM at 4 °C for 15 min and normalized by measuring protein absorbance at 280 nm. 10% of normalized lysates were saved for immunoblotting analysis. Pre-equilibrated Pierce anti-HA beads (Thermo Fisher Scientific 88837) were added to the remaining normalized whole cell extracts and incubated for 30 min to 1 h at 4 °C by vertical rotation. Protein bound beads were washed three times with wash buffer (100 mM Tris-HCl pH 8.0, 150 mM NaCl, 5% glycerol, 0.1% NP-40, 1 mM AEBSF, 1 μg/ml or 10 μM leupeptin, 1 μM pepstatin A) and eluted for immunoblotting analysis by boiling at 95 °C for 10 min in SDS sample loading buffer (20% glycerol, 0.01% bromophenol blue, 6% sodium dodecyl sulfate, and 120 mM Tris at pH 6.8) and then reduced with 10% β-mercaptoethanol. Samples containing either whole cell extracts or anti-HA immunoprecipitants were resolved by SDS-PAGE and transferred onto PVDF membranes. Membranes were blocked with either 5% milk or 5% BSA before blotting with primary antibodies against CIAPIN1 (Santa Cruz Biotechnology sc-271298, Lot #H2317), GLRX3 (Santa Cruz Biotechnology sc-100601 Lot#C1811), CIAO3 (Santa Cruz Biotechnology sc-514078 Lot #K1914 or Sigma Aldrich SAB4502760), NUBP1 (Santa Cruz Biotechnology sc-514175, Lot #0715), NUBP2 (Proteintech 15409-1-AP), MMS19 (Proteintech 16015-1-AP or 66049-1-IG), CIAO1 (Cell Signaling 87027S Lot:1), CIAO2B (Proteintech 20108-1-AP), CIAO2A (Proteintech 20776-1-AP), ERCC2 (Santa Cruz Biotechnology sc-101174, Lot #K0414), FTH1 (Cell Signaling #3998), FLAG (Sigma #F1804 and Proteintech), ISCU1/2 (Proteintech), HIF1α (Bethyl A300–286A), IRP2 (Santa Cruz Biotechnology sc-33682, Lot #B1116), FBXL5 (BioLegend Clone 3F5G12G9 or 10F4H9D12), GAPDH (Proteintech HRP-60004), and TUBA (Proteintech). Membranes were further blotted with HRP-labeled secondary antibodies before Pierce ECL Western Blotting Substrates (Thermo Fisher Scientific 32,106) or SuperSignal West Femto Maximum Sensitivity Substrate (Thermo Fisher Scientific 34,096) were applied. Membranes were visualized on exposed film or with the iBright Imaging System. Densitometric analysis of blots was carried out with ImageJ (32).

### Preparation of peptide standard

HEK293 cells were resuspended in lysis buffer (100 mM Tris-HCl pH 8.0, 8M urea, 1 mM DTT, 1 mM AEBSF, 1 μg/ml or 10 μM leupeptin, 1 μM pepstatin A and 1× phosphatase inhibitor cocktail). Protein abundance was estimated using absorbance at 280 nm. Protein solution was reduced with 5 mM Tris (2-carboxyethyl) phosphine, alkylated with 10 mM iodoacetamide, digested with trypsin overnight, and desalted with C18 cartridge. Dried peptides (51 μg)were resuspended in 102 μl 5% formic acid (Thermo Fisher Scientific 85,178) to make 500 ng/μl peptide standard, which was further diluted to 250 ng/μl, 125 ng/μl, 62.5 ng/μl with 5% formic acid.

### Affinity purification of protein complexes

Cell pellets from five 15 cm plates were resuspended in lysis buffer (100 mM Tris-HCl pH 8.0, 150 mM NaCl, 5% glycerol, 0.1% NP-40, 2 mM MgCl_2_, turbo nuclease, 1 mM DTT, 1 mM AEBSF, 1 μg/ml or 10 μM leupeptin, 1 μM pepstatin A). Lysates were vertically rotated for 30 min at 4 °C, cleared by centrifugation at 13,200 RPM at 4 °C for 15 min, and normalized using protein absorbance at 280 nm. Pre-equilibrated anti-HA beads (100 μl) (ThermoFisher Scientific 88,837) or 150 μl of EZview Red Anti-HA Affinity Gel (Sigma-Aldrich E6779) were added to the normalized whole cell extracts and incubated for 1 h at 4 °C by vertical rotation. Protein bound beads were washed three or five times with wash buffer (100 mM Tris-HCl pH 8.0, 150 mM NaCl, 5% glycerol, 0.1% NP-40, 1 mM AEBSF, 1 μg/ml or 10 μM leupeptin, 1 μM pepstatin A) and 1 or 2 additional time with clean wash buffer (100 mM Tris-HCl pH 8.0, 150 mM NaCl, 5% glycerol). Proteins were eluted in glycine (0.1 M, pH 2). Eluates were neutralized by Tris, and NaCl was added to a final concentration of 150 mM. 4× volume cold acetone was used to precipitate proteins at −20 °C for 2 h or overnight. Proteins were pelleted at 15000*g* for 25 min, and after discarding the supernatant, the protein pellet was washed by an additional 500 μl of pure acetone. The protein pellet was dried and resuspended in digestion buffer (8M Urea in 100 mM Tris pH 8.5), reduced with 5 mM Tris (2-carboxyethyl) phosphine, alkylated with 10 mM iodoacetamide, and digested with Lys-C and trypsin. Digestion was quenched by addition of formic acid to a final concentration of 5%. Digested peptides were desalted with C18 tip (ThermoFisher Scientific 87,784) and resuspended in 5% formic acid for subsequent analysis by LC/MS.

### Proteomic characterization of interactome

Peptides from purified protein complexes were analyzed on a Thermo Scientific Fusion Lumos Tribrid Mass Spectrometer after chromatographic separation. A Dionex UltiMate 3000 nanoLC system was used to deliver the chromatographic gradient onto an in-house packed 75 μm by 25 cm column composed of ReproSil-Pur C18 (r119.aq.0001). Columns were washed with buffer R1 (60% acetonitrile, 20% 2-propanol and 20% water) and equilibrated in buffer A (1% 0.1% formic acid and 3% DMSO in water) before sample loading. Gradient started with 99% buffer A (1% 0.1% formic acid and 3% DMSO in water) and 1% buffer B (0.1% formic acid and 3% DMSO in acetonitrile) at a flow rate of 400 nl/min. At the flow rate of 200 nl/min, buffer B increased to 5.5% within the next 5 min, to 27.5% in the subsequent 123 min, to 35% in the next 7 min, rapidly to 80% over 1 min, held at 80% for 2 min, and dropped back to 1% over the next 2 min. A 2200 V voltage was applied to ionize peptides. Samples were analyzed using data-dependent acquisition (DDA) where a full MS scan was acquired every 3 s at resolution of 120,000 with scan range set to 400 to 1600 m/z. Ions with charge states between 2 and 6 and an intensity greater than 4.0e3 were selected for fragmentation by quadrupole using a 1.6 m/z isolation window. Dynamic exclusion was set at 25 s. MS/MS spectra were collected using 35% collision energy at a resolution of 15,000.

### Database search for identifications

MaxQuant (version 1.6.10.43 or 2.0.3.0) with the built-in Andromeda algorithm was used to search proteomic data against the EMBL-EBI Human reference proteome (UP000005640_9606, updated in April 2019) containing 20,874 proteins with common contaminants appended (33). The following search parameters were used: peptide tolerance of 20 ppm for first search and 4.5 ppm for main search; fragment ion tolerance of 20 ppm; peptides containing fixed carbamidomethyl modification on cysteines with maximum five modifications per peptide in including variable methionine oxidation and protein N-terminus acetylation; digestion specific for trypsin and Lys-C with at most two missed cleavages; label-free quantification was enabled as needed with only unique peptides used for quantification. False detection rates were evaluated through a target decoy–based approach and filtered at 1% at both peptide spectrum match level and protein level. MS/MS spectra for proteins identified by only one unique peptide are shown in Fig. S1. MS1 level intensities from DDA experiments were calculated by MaxQuant. Changes in protein abundance were calculated using artMS with integrated MSstats package (34, 35). A linear mixed model was used to determine proteins with differential abundance.

### Development and analytical validation of the targeted MS assay/measurement for Fe-S–related proteins

For the targeted proteomics assay, candidate proteotypic peptides were selected from unbiased data-dependent analyses using criteria described by Rauniyar (36). Briefly, peptides must be unique to the human proteome, 7 to 20 amino acids in length, and lack missed cleavage sites. Preference was also given to peptides lacking methionine, cysteine, and tryptophan which are susceptible to oxidation. These candidate peptides were then targeted using PRM in samples derived from both whole cell lysates and immunoprecipitates. Chromatography and instrument settings were identical to unbiased proteomic analyses except as indicated below. For whole cell lysate experiments, settings included an isolation window of 0.7 m/z, HCD activation with 35% collision energy, and an orbitrap resolution at 30,000. Scan range mode was set to “Auto” with standard AGC target. Maximum injection time was set to “Dynamic” with at least 10 points across the peak. Data type was set to “Centroid”. For quantification of CIAO3 interactions, settings for targeted acquisition are similar except that isolation window was set to 1.6 m/z, maximum injection time was set to 54 ms, and full MS scan was acquired.

For analysis of PRM experiments, a spectral library was first generated using peptide spectrum matches from DDA acquisition of affinity purified protein complexes associated with 3HA-3FLAG-NUBP2 and His-HA-StrepII-CIAO1. Product ion chromatograms were then extracted using Skyline (20.2.0.343) (37). Extracted ion chromatograms were carefully inspected to ensure (1) co-elution of all fragment ions used for subsequent quantitation, (2) mass accuracy of the measured precursor and fragment ions relative to their theoretical masses, (3) dot product of the acquired spectra relative to its match in the spectral library, (4) differences in the observed *versus* expected retention times for each peptide after retention time alignment, and (5) reproducibility across multiple replicates. A high-quality list of quantotypic peptides was generated based on these data and is shown in Table S2. All PRM data used for assay validation have been added to Panorama Public with access URL https://panoramaweb.org/eGV5lu.url.

### Experimental design and rationale

For comparative targeted experiments, two biological replicates were performed to ensure consistency of observations. Negative controls that did not contain the targeted proteins were employed to ensure specificity. Precursors with a minimum of three transitions without interferences were manually selected to generate quantitative information. MSstats, an R package developed for statistical analysis and relative quantification of mass spectrometry-based proteomics was used to determine protein abundances and significant changes across conditions (35). Protein intensities were estimated using the summary method of Tukey’s median polish and normalized to bait protein by selecting peptides from CIAO3 as global standards. Significant changes in protein abundance were determined using a family of linear mixed-effects models. Adjusted *p*-values were calculated, and changes were considered significant if adjusted *p* < 0.05.

### Homology modeling of human CIAO3

Swiss-Model was used for homology modeling of human CIAO3 based on the structure of Fe-only hydrogenase (1FEH) (31, 38).

## Data availability

All relevant raw files for this study have been deposited into MassIVE data repository (MassIVE MSV000088394, PXD029770, https://doi.org/10.25345/C5T85M) and Panorama Public (PXD033557).

## Supporting information

This article contains supporting information.

## Conflict of interest

The authors declare that they have no conflicts of interest with the contents of this article.

## Supporting information

SupportingInformation

Supplemental TableS1

Supplemental TableS2

Supplemental TableS3

Supplemental TableS4
