# Supplementary material for: Iron-regulated assembly of the cytosolic iron–sulfur cluster biogenesis machinery
Source: J Biol Chem. 2022 May 30;298(7):102094. doi: 10.1016/j.jbc.2022.102094 (PMC9243173; doi:10.1016/j.jbc.2022.102094)
Supplement: SupportingInformation [file mmc1.docx]

**Supporting Information**

**Iron-Regulated Assembly of the Cytosolic Iron-Sulfur Cluster Biogenesis Machinery**

Xiaorui Fan (范 潇蕊), William D. Barshop, Ajay A. Vashisht, Vijaya Pandey, Stephanie Leal, Shima Rayatpisheh, Yasaman Jami-Alahmadi, Jihui Sha, James A. Wohlschlegel

Material included:

**Figure S1. Annotated MS/MS spectra for proteins identified with a single peptide.** (Related to Fig. 1B and 1D)

(A) DSLVLLGR for CIAO1 identification in Fig. 1B

(B) DLEDQEQLLR for POLD1 identification in Fig. 1B

(C) SEGGEDYTGATVIEPLK for POLD1 identification in Fig. 1D

**Supplemental Table Legends (Table S1 to S4; Excel files)**

**Figure S1. Annotated MS/MS spectra for proteins identified with a single peptide.**

**
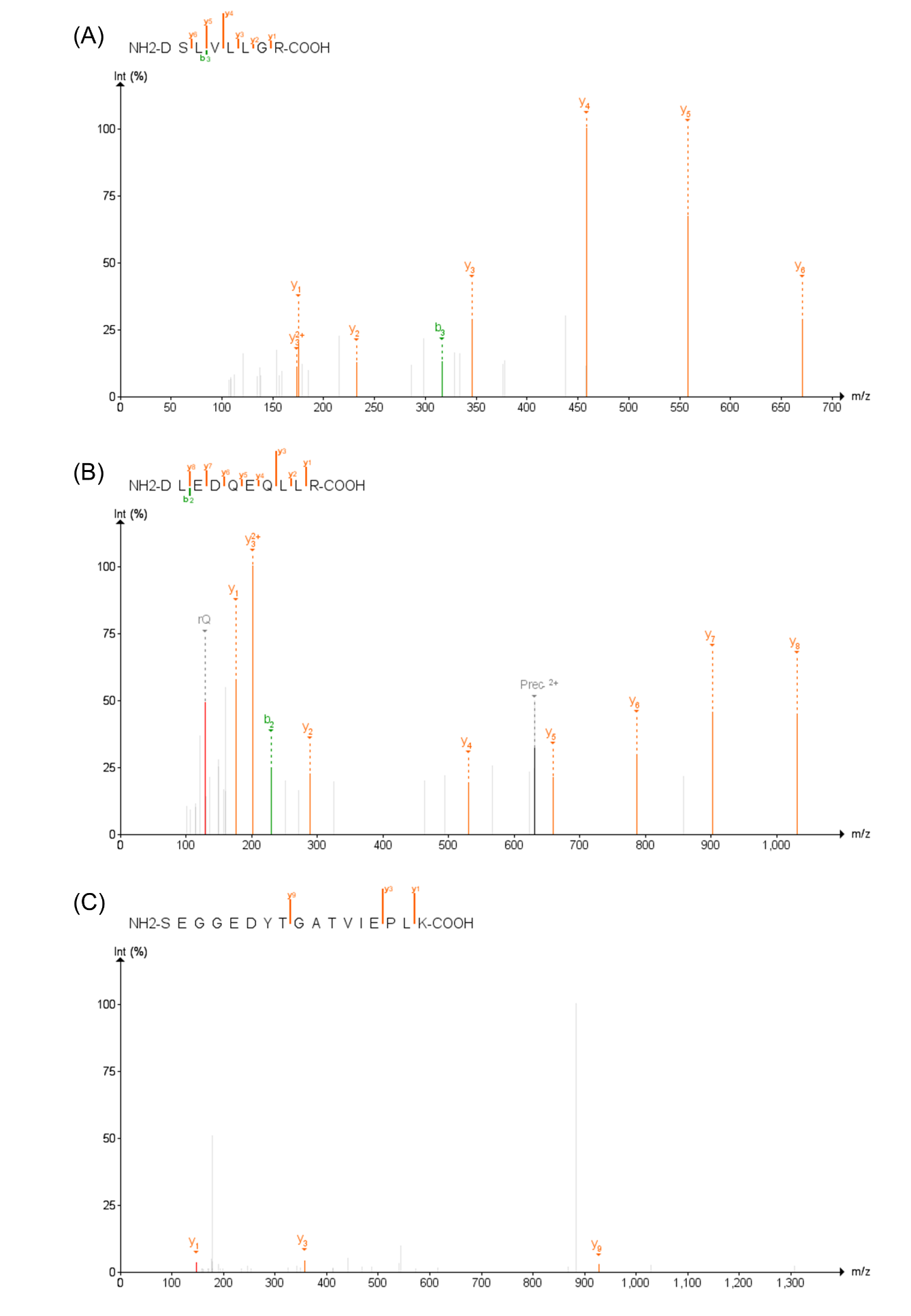
**

**Table S1. Proteins identified by data-dependent acquisition of NUBP2 and background immunoprecipitates. (**Related to Fig. 1B)

Flp-In 293 cells were cultured either stably expressing 3HA-3FLAG tagged NUBP2 (NUBP2) or with no ectopic protein (BG). Anti-HA immunoprecipitation was performed with cell lysates and the immunoprecipitates were processed for bottom-up proteomic analysis. Two technical replicates were performed. For each identified protein, the total number of unique peptides (combined from both technical replicates), protein sequence coverage, intensity and Q-value were reported in this table.

**Table S2. List of monitored peptides.** (Related to Fig. 1C, 1D, 2B and 4B to 4D)

This list includes mass-to-charge and charge of all precursors monitored. In the experiment identifying proteins that are associate NUBP2 (Fig. 1D), a subset of precursors was monitored to increase the sensitivity for the CIA targeting complex and substrates.

**Table S3. Proteins detected by parallel reaction monitoring in NUBP2 and background immunoprecipitates. (**Related to Fig. 1D)

NUBP2 immunoprecipitate (NUBP2) or immunoprecipitate from background cells (BG) were subject to analysis by parallel reaction monitoring. Precursors monitored were provided in Table S2. Two biological replicates were analyzed. The table includes the number of unique peptides identified in each protein, sequence of peptides identified and Q-value of each protein.

**Table S4. Iron-regulated CIAO3 interactions. (**Related to Fig. 2A)

Flp-In 293 cells stably expressing 3HA-3FLAG tagged CIAO3 (CIAO3) or background cells (BG) were treated with either FAC or DFO to stimulate a high or low iron environment. Anti-HA immunoprecipitation was performed and tryptic-digested immunoprecipitates were analyzed by data-dependent acquisition followed by intensity-based label-free quantification. The number of unique peptides, sequence coverage, Q-value and protein intensity were reported for each protein. The MSstats comparison of CIAO3-interacting proteins between the high and low iron conditions was also provided. This data was used for plotting Fig. 2A. Proteins were omitted from the list that were not significantly enriched for interacting with CIAO3.
